# Persistence rates and medical costs of biological therapies for psoriasis treatment in Japan: a real-world data study using a claims database

**DOI:** 10.1186/s12895-018-0074-0

**Published:** 2018-07-11

**Authors:** Rosarin Sruamsiri, Kosuke Iwasaki, Wentao Tang, Jörg Mahlich

**Affiliations:** 1 Health Economics, Janssen Pharmaceutical KK, 5-2, Nishi-kanda 3-chome Chiyoda-ku, Tokyo, 101-0065 Japan; 2 Center of Pharmaceutical Outcomes Research, Naresuan University, Phitsanulok, Thailand; 3 Milliman, Tokyo, Japan; 4 Health Economics and Outcomes Research, Janssen-Cilag GmbH, Johnson & Johnson Platz 1, Neuss, 41470 Germany; 5 Düsseldorf Institute for Competition Economics (DICE), University of Düsseldorf, Düsseldorf, Germany

**Keywords:** Psoriasis, Biological therapy, Persistence, Medical costs, Real-world data, Claims database

## Abstract

**Background:**

Biological therapies (BTs) including infliximab (IFX), adalimumab (ADL), secukinumab (SCK) and ustekinumab (UST) are approved in Japan for the treatment of psoriasis. Although the persistence rates and medical costs of BTs treatment have been investigated in multiple foreign studies in recent years, few such studies have been conducted in Japan and the differences between patients who adhered to treatment and those who did not have not been reported. This study is aimed at investigating the persistence rates and medical costs of BTs in the treatment of psoriasis in Japan, using the real-world data from a large-scale claims database.

**Methods:**

Claims data from the JMDC database (August 2009 to December 2016) were used for this analysis. Patient data were extracted using the ICD10 code for psoriasis and claims records of BT injections. Twelve-month and 24-month persistence rates of BTs were estimated by Kaplan-Meier methodology, and 12-month-medical costs before and after BT initiation were compared between persistent and non-persistent patient groups at 12 months.

**Results:**

A total of 205 psoriasis patients treated with BTs (BT-naïve patients: 177) were identified. The 12-month/24-month persistence rates for ADL, IFX, SCK, and UST in BT-naïve patients were 46.8% ± 16.6%/46.8 ± 16.6%, 53.0% ± 14.9%/41.0% ± 15.5%, 55.4%/55.4% (95% CI not available) and 79.4% ± 9.9%/71.9% ± 12.2%, respectively. Statistically significant differences in persistence were found among different BT treatments, and UST was found to have the highest persistence rate. The total medical costs during the 12 months after BT initiation in BT-naïve patients were (in 1000 Japanese Yen): 2218 for ADL, 3409 for IFX, 465 for SCK, 2824 for UST (average: 2828). Compared with the 12-month persistent patient group, the total medical costs in the persistent group was higher (Δ:+ 118), but for some medications such as IFX or UST cost increases were lower for persistent patients.

**Conclusions:**

UST was found to have the highest persistence rate among all BTs for psoriasis treatment in Japan. The 12-month medical costs after BT initiation in the persistent patient group may not have increased as much as in the non-persistent patient group for some medications.

**Electronic supplementary material:**

The online version of this article (10.1186/s12895-018-0074-0) contains supplementary material, which is available to authorized users.

## Background

Psoriasis is a chronic autoimmune disease characterized by inflammatory plaques of the skin and known to have a significantly negative impact on patients’ quality of life (QoL) [1, 2]. The estimated prevalence of psoriasis in Japan has been reported in previous studies to be 0.34%–0.44% in 2010–2012, and this is expected to increase annually [3, 4].

Biological therapies (BTs) using monoclonal antibodies have been developed to treat psoriasis and to relieve psoriatic symptoms while improving patients’ QoL. In Japan, four BTs are for treating psoriasis: infliximab (IFX) and adalimumab (ADL) were approved in 2010, ustekinumab (UST) was approved in 2011, and secukinumab (SCK) was approved in 2014.

Despite the documented benefits of BTs in the treatment of psoriasis, persistence to BT (i.e., the duration of time from initiation to discontinuation of therapy) may vary considerably depending on country, patient characteristics and specific drug used for treatment. The medical costs of BT psoriasis treatment in a hospital setting also need to be investigated.

Multiple previous studies analyzing the persistence rate of BTs in psoriasis patients have been conducted in the European Union (EU) [5–9] and the United States (US) [10–13], indicating the related high level of interest and activity. These studies used patient registries, claims databases, or hospital-based patient cohorts. Overall, the EU studies reported higher 12-month persistence rates for BTs (53%–90%) than US studies (25%–66.7%), suggesting a certain level of outcome heterogeneity among different countries.

Consequently, results from studies in other countries may not reflect the experience in Japan. Few previous studies have been conducted to investigate the use of BT in psoriasis in Japan. Umezawa (2013) [14] analyzed the persistence to ADL, IFX, and UST, using a patient cohort from Jikei University School of Medicine. However, it is not clear to what degree the results are representative of the general population of psoriasis patients across Japan.

Therefore, this study is aimed at investigating the BT psoriasis treatment persistence rates as well as comparing medical costs between patients who were persistent with BT treatment and those who did not, based on data from a large-scale, real-world (RW) claims database in Japan.

## Methods

### Data source

We utilized the JMDC (Japan Medical Data Center Co., Ltd.) database which contains claims and annual examination data of employees and their dependents from the employees’ insurance program in Japan [15]. This database includes approximately 2.7 million members and represents approximately 2.1% of the total Japanese population. The JMDC database has been used to investigate a wide range of conditions in Japan such as schizophrenia, rheumatoid arthritis, or cardiovascular disease [16–18].

The time span of our analysis was August 2009 to December 2016. As patient data were anonymized by the database provider, no informed consent was necessary.

### Study population and study design

For the analysis of persistence rates, the study population was identified based on the International Classification of Diseases, 10th revision (ICD-10) [19] and claims records of BT injections. Patients who had both a diagnosis of psoriasis as ICD 10: L40 at any time and claims records that documented the receipt of a BT via injection at any location (i.e. at a clinic or as self-injection at home) were selected for analysis. Patients with a diagnosis of rheumatoid arthritis, inflammatory bowel disease, ankylosing spondylitis, and juvenile arthritis were excluded. ICD-10 codes for these diseases are listed in Appendix A (see Additional file 1).

Among the patients selected for analysis, BT-naïve patients were further identified as those who had no record of BT prescription within one year before the BT index date in the database. BT-naïve patients were used for primary analysis of persistence rates, while the total patient group, including both BT-naïve and BT-experienced patients, was also used for secondary analysis.

For the analysis of medical costs, the index date was defined as the first claim for a BT. Among patients selected for the analysis of persistence rates, only those who had at least 24 months of follow-up data (12 months both pre- and post-BT initiation) in the database were eligible for a calculation and comparison of medical costs pre- and post- BT initiation.

Similar to the analysis for persistence rates, the BT-naïve patient group was used for primary analysis of medical costs while the total patient group, including both BT-naïve and BT-experienced patients, was also used for secondary analysis.

### Outcomes

**Fig. 1:**
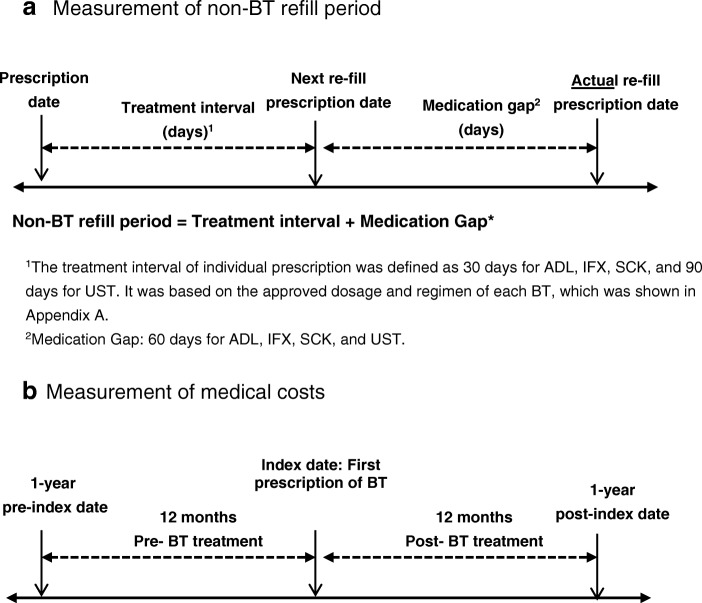
Measurement of non-BT refill period and medical costs. Legends: **a** Measurement of non-BT refill period. ^1^The treatment interval per individual prescription was defined as 30 days for ADL, IFX, SCK, and 90 days for UST. It was based on the approved dosage and treatment regimen for each BT, which is shown in Appendix A. ^2^Medication Gap: 60 days for ADL, IFX, SCK, and UST. **b** Measurement of medical costs

Primary outcome of this study was the persistence rate for BT over time. We used the Kaplan-Meier method to estimate the 12-month and 24-month persistence rates for ADL, IFX, SCK, and UST treatment in the BT-naïve group and the total patient group. The persistence period was defined as the time from treatment initiation (index date) until discontinuation of the index BTs (Fig. 1a).

Patients were categorized as discontinuing the index BT based on whichever of the following occurred first: (1) a period of 90 consecutive days without the index BT (non-BT refill period) for ADL, IFX, SCK, or 150 days for UST was found; or (2) the patient switched from the index BT to other treatment(s) during follow-up. This definition of persistence was consistent with the one employed in other studies with BTs for psoriasis treatment using claims data [10, 12].

The non-BT refill period was defined as the sum of the treatment interval and medication gap. The treatment interval was defined as 30 days for ADL, IFX, SCK, and 90 days for UST, based on the approved dosage and regimen of each BT (see Appendix B, Additional file 1). A medication gap of 60 days was used for all BTs.

In addition, for patients who have a 12 month-follow-up period both pre- and post- BT initiation, the persistence rates (number of persistent patients during 12 months after BT initiation/number of total patients) were calculated. The definition of the non-BT refill period was the same as the one used for the Kaplan-Meier analysis. The medication gap used in the base case was 60 days, but was varied in the sensitivity analyses. The gap definitions used in the base case and sensitivity analysis are presented in Appendix C (see Additional file 1).

Only patients who had at least 12-months of follow-up data pre- and post- BT initiation were selected for the analysis of medical costs. Medical costs were determined and compared for the 12 months before the index date (first initiation of BTs) and the 12 months after the index date in the BT-naïve/total patient group (Fig. 1 b).

These patients were split into two more groups. Patients who continued BT for a 12-month period were allocated to the persistence group, while others were allocated to the non-persistence group. The differences in medical costs between these two groups were also analyzed.

The analysis of medical costs included the following items: outpatient medical costs, inpatient medical costs, costs of drugs other than BTs, and overall costs. Costs of BTs were included in outpatient medical costs or inpatient medical costs. All costs were calculated based on Japanese Yen (JPY).

### Statistical analysis

Descriptive statistics were used to analyze the characteristics of patients receiving BTs, along with the medical costs they caused pre- and post- BT initiation.

Kaplan-Meier curves were plotted to show the persistence to different BTs. The log-rank and Wilcoxon rank sum tests were used to assess the differences in persistence rates for different BTs in the whole group and BT-naïve patient groups. A *p* value of ≤0.05 was considered statistically significant. The analysis was undertaken using SAS software (ver. 9.4; SAS Institute Inc. Cary, NC, USA).

## Results

### Study population

**Fig. 2:**
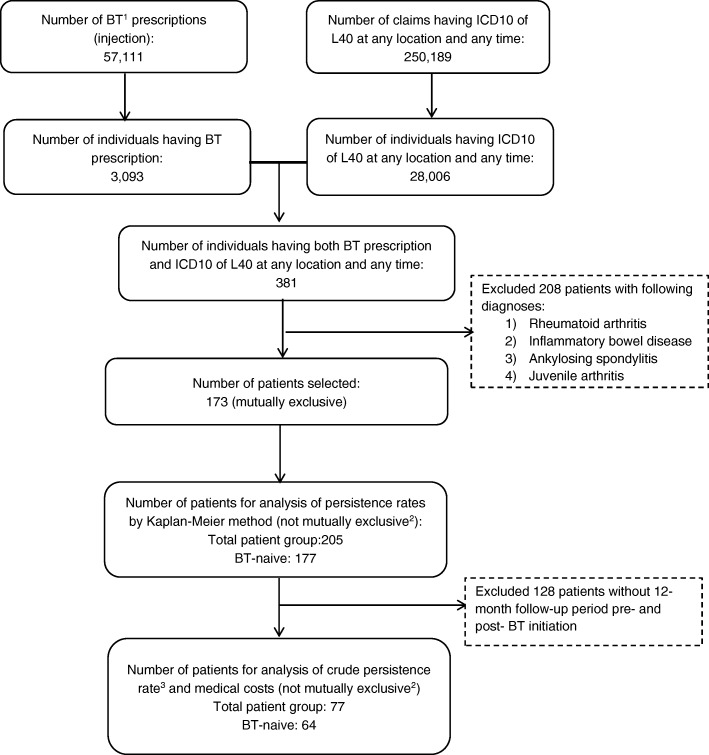
Cascade figure of patient flow. Legends: ^1^BT: biological therapies; ^2^for the purpose of analysis, multiple cycles of any BT treatment administered to one patient were counted as different patients. ^3^number of persistent patients during 12-month after BT initiation/number of total patients

The patient flow diagram for the analysis is shown in Fig. 2. 28,006 patients with a diagnosis of psoriasis were identified from 250,189 claims with ICD-10: L40. A total of 3093 patients who used BTs were identified from 57,111 claims of BT injections. Overall, 381 patients who used BTs and had a diagnosis of psoriasis were extracted. A total of 208 patients who had received a diagnosis of rheumatoid arthritis, inflammatory bowel disease, ankylosing spondylitis, or juvenile arthritis were excluded. In the end, 173 patients were selected for the analysis.

Table 1 shows the patient characteristics of each treatment group in total and stratified by BT-naïve/experienced status. Patients who had received more than one BT in the past were counted in each separate BT group. By counting each patient separately for each BT received, a total of 205 patients were identified for analysis, and patients in each BT group were not mutually exclusive. Among them, 177 patients were BT-naïve, while the other 28 patients were BT-experienced. The mean age of the total, BT-naïve, and BT-experienced patient group was 47.1 years, 47.3 years, and 46.2 years, respectively. The percentage of female patients in the total, BT-naïve, and BT-experienced group was 18.1%, 19.8%, and 7.1%, respectively.

**Table 1:** Demographics of total patient population

	Total					BT-naïve					BT-experienced				
	ADL	IFX	SCK	UST	Sub total	ADL	IFX	SCK	UST	Sub total	ADL	IFX	SCK	UST	Sub total
Number of patients	42	52	21	90	205	37	48	13	79	177	5	4	8	11	28
Average Age	50.0	46.2	45.4	46.8	47.1	50.9	46.0	46.2	46.5	47.3	43.4	47.5	44.3	48.4	46.2
% Female	7.1%	21.2%	33.3%	17.8%	18.1%	8.1%	20.8%	46.2%	20.3%	19.8%	0.0%	25.0%	12.5%	0.0%	7.1%

*ADL* Adalimumab, *IFX* Infliximab, *SCK* Secukinumab, *UST* Ustekinumab

In Table 1, the respective numbers of total, BT-naïve and BT-experienced patients for each of the specific treatment groups are reported as well and were as follows: 42, 37, and 5 in the ADL group; 52, 48, and 4 in the IFX group; 21, 13, and 8 in the SCK group; and 90, 79, and 11 in the UST group. Among the 205 patients who received BTs, 77 patients provided 12-month data both pre- and post- BT initiation; 42 of 77 patients continued the BT they initiated for 12 months, while the other 35 patients discontinued within 12 months of starting treatment.

Table 2 shows the prevalence of comorbidities in the overall group of 205 patients. Hyperlipidemia and hypertension were the most common co-morbidities.

**Table 2:** Comorbidity characteristics of total patient population

	ADL	IFX	SCK	UST	Total
Number of patients	42 (100%)	52 (100%)	21 (100%)	90 (100%)	205 (100%)
Obesity	0 (0%)	0 (0%)	0 (0%)	1 (1.1%)	1 (0.5%)
Diabetes without complication/comorbidity	2 (4.8%)	4 (7.7%)	0 (0%)	6 (6.7%)	12 (5.9%)
Diabetes with complication/comorbidity	0 (0%)	2 (3.8%)	1 (4.8%)	4 (4.4%)	7 (3.4%)
Hypertension	6 (14.3%)	11 (21.2%)	4 (19.0%)	28 (31.1%)	49 (23.9%)
Hyperlipidemia	8 (19.0%)	13 (25.0%)	5 (23.8%)	20 (22.2%)	46 (22.4%)
Subsequent/old myocardial infarction	0(0%)	1 (1.9%)	0(0%)	1 (1.1%)	2 (1.0%)
Heart failure	2 (4.8%)	4 (7.7%)	0 (0%)	8 (8.9%)	14 (6.8%)

*ADL* Adalimumab, *IFX* Infliximab, *SCK* Secukinumab, *UST* Ustekinumab, *DLMOL* Disorders of lipoprotein metabolism and other lipidemias

### Persistence rates

**Fig. 3:**
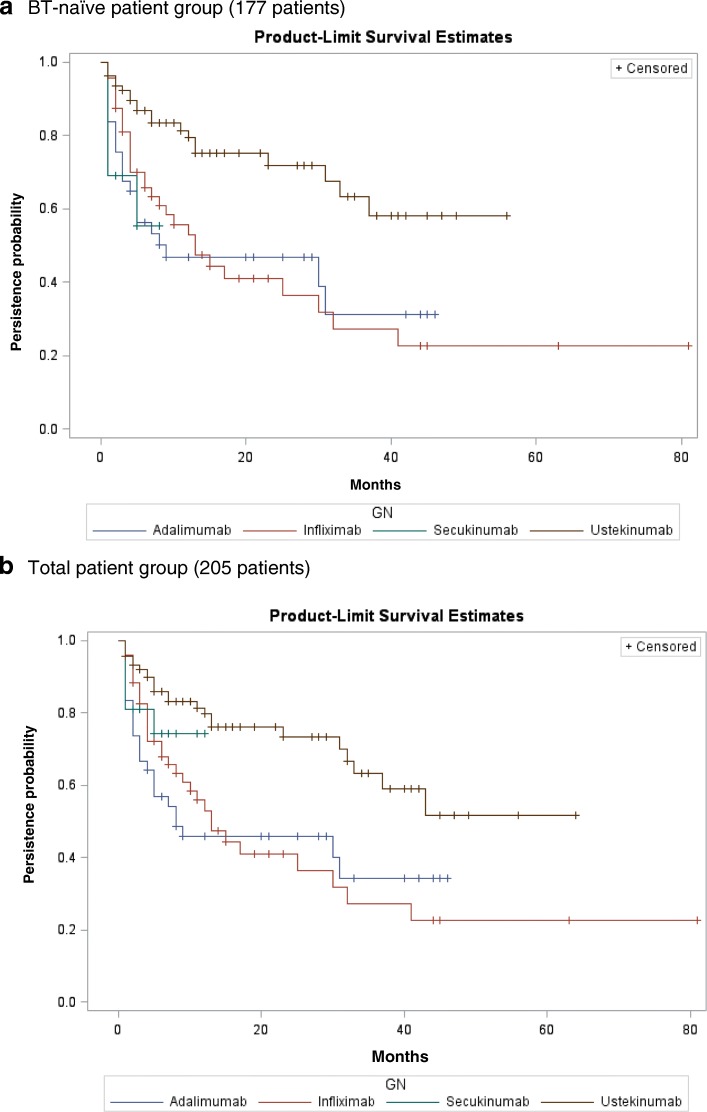
Kaplan-Meier Curves of BT persistence in the BT-naïve and the total patient group Legends: **a** BT-naïve patient group (number: 177 patients). **b** Total patient group (number: 205 patients).

Figure 3 presents the Kaplan Meier curves, using a non-BT refill period of 90 days for ADL, IFX and SCK, and 150 days for UST, for both the BT-naïve patient group and the total patient group.

The 12/24-month persistence rates for each BT are shown in Table 3.

**Table 3:** 12/24-month persistence rates and 95% confidence intervals (CI) for BTs psoriasis therapy

	Total				BT-naïve			
	ADL	IFX	SCK	UST	ADL	IFX	SCK	UST
Number of patients	42	52	21	90	37	48	13	79
12-month persistence rate ± 95% CI	45.9% ± 15.5%	53.1% ± 14.7%	74.2% (95% CI not available)	79.8% ± 9.1%	46.8% ± 16.6%	53.0% ± 14.9%	55.4% (95% CI not available)	79.4% ± 9.9%
24-month persistence rate ± 95% CI	45.9 ± 15.5%	41.1% ± 15.4%	74.2% (95% CI not available)	73.4% ± 10.9%	46.8 ± 16.6%	41.0% ± 15.5%	55.4% (95% CI not available)	71.9 ± 12.2%
*p*-value of log-rank test (versus UST)	< 0.001	< 0.001	0.254	–	< 0.001	< 0.001	0.006	–
*p*-value of wilcoxon test (versus UST)	< 0.001	0.001	0.163	–	< 0.001	0.001	0.004	–

*ADL* Adalimumab, *IFX* Infliximab, *SCK* Secukinumab, *UST* Ustekinumab. 95% CI of SCK was not available because of too small number of patients

In the total patient group, the 12-month persistence rates (±95% CI) for ADL, IFX, SCK, and UST were 45.9% ± 15.5%, 53.1% ± 14.7%, 74.2% (95% CI for SCK not available) and 79.8% ± 9.1%, respectively. The 24-month persistence rates were 45.9% ± 15.5% (ADL), 41.1% ± 15.4% (IFX), 74.2% (95% CI for SCK not available), and 73.4% ± 10.9% (UST). Statistically significant differences were found in the persistence rates among different BTs, either by log-rank test or Wilcoxon test (both *p* < 0.001). UST was found to have a significantly higher persistence rate in the pairwise comparisons with ADL and IFX (all *p* values ≤0.001 in either the log-rank test or Wilcoxon test), while no statistically significant difference was found between UST and SCK (log-rank test *p* = 0.254; Wilcoxon test *p* = 0.163). The proportions of patients who maintained persistent during the 12-month period after initiating BT - as relative to the total number of patients calculated in the base case and sensitivity analyses - are shown in Appendix D (see Additional file 1).

For BT-naïve patients, the 12-month persistence rates ±95% confidence intervals (CI) for ADL, IFX, SCK, and UST were 46.8 ± 16.6, 53.0 ± 14.9%, 55.4% (95% CI for SCK not available), and 79.4% ± 9.9%, respectively. The 24-month persistence rates were 46.8% ± 16.6% (ADL), 41.0% ± 15.5% (IFX), 55.4% (95% CI for SCK not available), and 71.9% ± 12.2% (UST). Statistically significant differences were found in the persistence rates among different BTs, either by the log-rank test or Wilcoxon test (both *p* < 0.001). UST was found to have a significantly higher persistence rate not only in the pairwise comparisons with ADL and IFX (all *p* values ≤0.001 in either log-rank test or Wilcoxon test), but also in the pairwise comparisons with SCK (log-rank test *p* = 0.006, Wilcoxon test *p* = 0.004).

### Medical costs

Overall, 77 patients (BT-naïve patients: 64) providing data for the 12-month periods pre- and post-BT initiation were analyzed for medical costs. Table 4 outlines the medical costs pre- and post-BT treatment initiation in BT-naïve patients and the total patient group (all cost data presented in 1000 JPY units). Costs included inpatient medical costs, outpatient medical cost, costs of drugs other than BTs, and total costs. As only two patients with a secukinumab treatment fulfilled the inclusion criteria for cost analysis, we will not report the cost assessment for this treatment. Among all patients, 12-month total costs before biologic treatment initiation was ¥886,000 per patient. First year cost increase after initiation of biologic treatment was ¥1,907,000.

**Table 4:** Characteristics and medical costs of patients with 12 month-follow-up period pre- and post-BT initiation

		Total				BT-naïve			
		ADL	IFX	UST	Sub total	ADL	IFX	UST	Subtotal
Number of patients		18	20	37	77	14	19	30	64
Average Age		47.7	46.5	46.2	46.8	49.6	47.3	46.3	47.5
%Female		11.1%	20.0%	16.2%	15.6%	14.3%	21.1%	20.0%	18.8%
Cost (pre)		¥760	¥950	¥900	¥886	¥365	¥916	¥603	¥641
In 1000 JPY unit	IP	¥18	¥343	¥254	¥235	¥0	¥361	¥162	¥188
	OP	¥513	¥301	¥425	¥405	¥133	¥261	¥216	¥210
	RX	¥229	¥306	¥221	¥246	¥232	¥294	¥225	¥244
Cost increase		¥1538	¥2429	¥1865	¥1907	¥1853	¥2494	¥2221	¥2187
In 1000 JPY unit	IP	¥95	¥601	- ¥37	¥156	¥146	¥633	¥21	¥228
	OP	¥1077	¥1958	¥2011	¥1754	¥1379	¥1974	¥2296	¥1965
	RX	¥366	- ¥130	- ¥109	- ¥2	¥328	- ¥113	- ¥96	- ¥5

*ADL* Adalimumab, *IFX* Infliximab, *UST* Ustekinumab, pre:12 months pre-initiation, cost increase: increase from pre to post 12 months post-initiation, *JPY* Japanese Yen, *IP* inpatient medical costs, *OP* outpatient medical cost, *RX* costs of drugs other than BTs

Among the 64 BT-naïve patients, total medical cost per patient during the 12 months after BT initiation averaged 2828,000 JPY. Compared to the 12 months before BT initiation, the increase in medical costs was 2,187,000 JPY. Among the 64 BT-naïve patients, 33 patients (33/64, 51.6%) who continued BT treatment over 12 months constituted the persistent group, while the remaining 31 patients (31/64, 48.4%) who discontinued formed the non-persistent group. Tables 5 and 6 show the medical costs in the persistent and the non-persistent group.

**Table 5:** Characteristics and medical costs of patients in the persistent group

		Total				BT-naïve			
		ADL	IFX	UST	Sub total	ADL	IFX	UST	Sub total
Number of patients		6	6	29	42	4	6	23	33
Average Age		50.8	44.7	45.0	46.0	54.0	44.7	44.8	45.9
%Female		16.7%	16.7%	17.2%	16.7%	25.0%	16.7%	21.7%	21.2%
Cost (pre)		¥1092	¥1062	¥925	¥988	¥360	¥1062	¥597	¥653
In 1000 JPY unit	IP	¥55	¥603	¥315	¥340	¥0	¥603	¥201	¥250
	OP	¥876	¥398	¥418	¥473	¥263	¥398	¥211	¥251
	RX	¥161	¥61	¥192	¥175	¥97	¥61	¥186	¥152
Cost increase		¥1606	¥2246	¥1875	¥1883	¥2433	¥2246	¥2211	¥2244
In 1000 JPY unit	IP	- ¥55	- ¥174	- ¥147	- ¥140	¥0	- ¥174	- ¥98	- ¥100
	OP	¥1365	¥2446	¥2114	¥2054	¥2461	¥2446	¥2380	¥2402
	RX	¥295	- ¥26	- ¥91	- ¥30	- ¥27	- ¥26	- ¥71	- ¥57

*ADL* Adalimumab, *IFX* Infliximab, *UST* Ustekinumab, pre:12 months pre-initiation, cost increase: increase from pre to post 12 months post-initiation, *JPY* Japanese Yen, *IP* inpatient medical costs, *OP* outpatient medical cost, *RX* costs of drugs other than BTs

**Table 6:** Characteristics and medical costs of patients in the non-persistent group

		Total				BT-naïve			
		ADL	IFX	UST	Sub total	ADL	IFX	UST	Subtotal
Number of patients		12	14	8	35	10	13	7	31
Average Age		46.1	47.3	50.5	47.8	47.9	48.5	51.1	49.1
%Female		8.3%	21.4%	12.5%	14.3%	10.0%	23.1%	14.3%	16.1%
Cost (pre)		¥595	¥902	¥808	¥762	¥367	¥848	¥621	¥629
In 1000 JPY unit	IP	¥0	¥231	¥32	¥108	¥0	¥249	¥36	¥122
	OP	¥332	¥259	¥448	¥324	¥81	¥198	¥232	¥166
	RX	¥263	¥412	¥328	¥330	¥286	¥401	¥353	¥341
Cost increase		¥1504	¥2507	¥1828	¥1937	¥1620	¥2608	¥2254	¥2126
In 1000 JPY unit	IP	¥170	¥933	¥362	¥511	¥204	¥1005	¥414	¥577
	OP	¥932	¥1749	¥1638	¥1395	¥946	¥1756	¥2021	¥1499
	RX	¥402	- ¥175	- ¥172	¥31	¥470	- ¥153	- ¥181	¥50

*ADL* Adalimumab, *IFX* Infliximab, *UST* Ustekinumab, pre:12 months pre-initiation, cost increase: increase from pre to post 12 months post-initiation, *JPY* Japanese Yen, *IP* inpatient medical costs, *OP* outpatient medical cost, *RX* costs of drugs other than BTs

In the persistent group, the total medical costs during the 12 months after BT initiation averaged at 2,897,000 JPY. Compared with the 12 months before BT initiation the medical cost increase was 2,244,000 JPY on average. In the non-persistent group, total medical costs during the 12 months after BT initiation were 2,755,000 JPY on average. Compared with 12 months before BT initiation the increase in medical costs was 2,126,000 JPY.

The results of the medical cost comparison between the persistent and non-persistent groups are shown in Table 7. Only among patients receiving ADL the 12-month cost increase after BT initiation was smaller in the non-persistent than in the persistent group (Δ:-812). This difference was mainly due to the outpatient medical cost differences including drug costs for BTs (Δ:-1514). At the same time, costs of drugs other than BTs also showed differences (Δ: + 498). Among patients receiving IFX or UST the 12-month medical cost increase after BT initiation was larger in the non-persistent group than in the persistent group (Δ: + 362 for IFX, + 43 for UST). Similar results comparing the persistent and non-persistent groups were also found by the analysis of the total group of 77 patients. Compared with the persistent group, the 12-month medical costs after BT initiation in the non-persistent group decreased most for patients who received ADL (Δ:-599). Among patients receiving UST, a slight decrease was observed (Δ:-164). Among patients receiving IFX, 12-month medical costs after BT initiation increased for the non-persistent group (Δ: + 101).

**Table 7:** Comparison of medical costs of BT-naïve patients between the persistent (P) and non-persistent (NP) group

		ADL			IFX			UST			Total		
		P	NP	Δ	P	NP	Δ	P	NP	Δ	P	NP	Δ
Number of patients		4	10		6	13		23	7		33	31	
Cost (pre)		¥360	¥367	¥7	¥1062	¥848	- ¥214	¥597	¥621	¥24	¥653	¥629	- ¥24
In 1000 JPY unit	IP	¥0	¥0	¥0	¥603	¥249	- ¥354	¥201	¥36	- ¥165	¥250	¥122	- ¥128
	OP	¥263	¥81	- ¥182	¥398	¥198	- ¥200	¥211	¥232	¥21	¥251	¥166	- ¥85
	RX	¥97	¥286	¥189	¥61	¥401	¥340	¥186	¥353	¥167	¥152	¥341	¥189
Cost (increase)		¥2433	¥1621	- ¥812	¥2246	¥2608	¥362	¥2211	¥2254	¥43	¥2244	¥2126	-¥118
In 1000 JPY unit	IP	¥0	¥204	¥204	-¥174	¥1005	¥1179	-¥98	¥414	¥512	-¥100	¥577	¥677
	OP	¥2460	¥946	- ¥1514	¥2446	¥1756	-¥690	¥2380	¥2021	-¥359	¥2402	¥1499	-¥903
	RX	-¥27	¥471	¥498	-¥26	-¥153	-¥127	-¥71	-¥180	-¥109	-¥57	¥50	¥107

*P* persistent, *NP* non-persistent, Δ costs of NP minus costs of P, *ADL* Adalimumab, *IFX* Infliximab, *UST* Ustekinumab, pre:12 months pre-initiation, increase: increase from pre to post 12 months post-initiation, *JPY* Japanese Yen, *IP* inpatient medical costs, *OP* outpatient medical cost, *RX* costs of drugs other than BTs

## Discussion

### Persistence rates

Our analysis using RWD from Japan (both in the BT-naïve patient group and the total patient group) found the BT persistence rates among psoriasis patients to be higher than those reported in the US studies [10–12] but lower than the persistence rates reported in the EU studies [5–9, 20]. Regarding UST, the 12-month persistence rates reported in the two US studies by Chastek (2016) [10] and Gu (2016) [11] were 43.3 and 25%, respectively. These rates were obviously lower than those reported in the EU studies (78.6–90%) and the rates as per our analysis (79.4). The main reason for such differences may be that Chastek (2016) and Gu (2016) defined a non-BT refill period of 45 days, which was shorter than the non-BT refill periods used in the EU studies and our study. The defined length of the non-BT refill period was 90 days or 180 days in the EU studies, while it was 150 days for UST, and 90 days for the other BTs in our study. As UST’s approved label defines a longer interval between injections than the labels of other BTs, using a shorter non-BT refill period is considered to have a greater impact on UST’s persistence rate. Using different non-BT refill periods for different BTs as per each drug’s approved label, which were factored into our analysis of Japanese RWD data, is considered more appropriate for analyzing the persistence rate. This was also suggested in another US study by Doshi (2016) [12], which defined non-BT refill period as the sum of treatment interval as per regimen and a medication gap of 90 days for all BTs. The 12-month persistence rate for UST used by Doshi (2016) increased conspicuously to 65% compared with Chastek (2016) and Gu (2016). However, even the UST adherence rate reported by Doshi (2016) was still lower by comparison than the one reported in the EU studies and our study. This difference may be a result of the older patient group (mean age: 60.7 years) analyzed by Doshi (2016), compared with the EU studies and our study (mean age: approximately 50 years and younger). Other possible reasons such as differences in the health insurance systems, prescription preferences, and cultural factors were not covered in this study but may need to be discussed in future studies. Regarding ADL and IFX, the 12-month persistence rates demonstrated in our study were closer to the rates reported in the US studies and, by comparison, lower than those reported in the EU studies. Such differences were especially large among patients receiving ADL, so that our study found 12-month persistence rate for ADL of 46.8%, while that in the EU studies ranged from 64.6 to 79.7%. The 12-month persistence rate for IFX in our study was 53.0%, while that in the EU studies ranged from 63.6 to 70.9%. The causes of such differences with ADL and IFX treatment were unclear, as the possible differences between Japan and the EU with respect to treatment outcomes [21], incidence of anti-IFX and anti-ADL antibodies [22, 23], and prescription patterns of ADL and IFX were considerable. Moreover, the Japanese study by Umezawa (2013) reported much higher persistence rates than our study, namely 73.3, 79.7 and 96.7% for IFX, ADL and UST, respectively [14]. This study was a cohort study conducted in one hospital, while our study was based on a RWD database, which was considered as more representative of the patients with psoriasis across Japan, and the persistence rates of our study were closer to the reports from other studies.

### Medical costs

Although the psoriasis treatment outcomes were considered to have been improved by the introduction of BTs, the high costs of these drugs remain an important issue [24]. Several studies have discussed the costs of BTs and found a negative association between the patient motivation to initiate BT and its costs [25]. In general, it was evident that inpatient costs were generally higher after initiation of biologic therapy. Part of this cost increase might be due to hospitalizations for adverse events, e.g. infections, that patients experience when taking the new medication [26].

In our analysis of BT-naïve patients, the increases in the 12-month medical costs after BT initiation compared with those before BT initiation were highest in patients with IFX and lowest in patients with ADL. However, the 12-month medical costs after BT initiation were considered to be influenced by the persistence rates for the different BTs. When comparing the 12-month medical costs after BT initiation in the persistent and non-persistent groups, we found the most conspicuous cost decrease for ADL in the non-persistent group compared with the persistent group, namely from 2793 to 1988 JPY (Δ: -28.8%). Conversely, the costs for UST and IFX increased in the non-persistent group compared with the persistent group (Δ: + 2.4% for UST, + 4.5% for IFX). This finding suggested that the medical costs for patients who discontinued BTs within 12 months may not decrease by much but instead have the potential to increase compared to the costs for patients who were persistent with the BTs. Similar analyses of a period longer than 12 months are expected to be performed in future studies. The costs for drugs other than BTs increased for all non-persistent patients compared with the costs for persistent patients (Δ: + 687 for ADL; + 213 for IFX; and + 58 for UST), suggesting an increase in alternative treatments after patients discontinued BTs. Moreover, some patients were found to discontinue BTs due to the adverse events they experienced during the treatment. The treatment for adverse events may also contribute in part to the increase in drug costs not related to BTs.

This study is not an economic cost effectiveness evaluation of specific treatments, which was performed elsewhere. According to Igarashi et al. (2013), annual costs (2013) for ADL were 23,995 USD in the first and 23,107 USD in the second year. For IFX the costs were 38,860 USD and 32,593 USD, for UST they were 26,660 and 23,087. PASI 75 response rates were 83% for IFX, 74% for UST, and 59% for ADL. In the first year of induction treatment, the lowest cost per responder was for UST, followed by ADL and IFX. In the subsequent year of maintenance treatment, the cost per responder for UST remained the lowest [27].

A more recent study of Imafuku et al. (2018), however, reported annual treatment costs amounting to 15,668 USD for ADL (40 mg), 25,522 USD for IFX, 19,541 USD for UST (45 mg), and 10,423 USD for SEC. Assumed PASI 75 response rates in that study were 64% for IFX, 64% for UST, and 74% for ADL. Costs per responder were therefore lowest for ADL and SCK [28].

### Limitations

There are several limitations to this study. First of all, evidence quality generated from the claims data analyses is generally limited due to the limited parameters available in the claims database. In this study, we were unable to filter by disease severity and disease activity of psoriasis at the time of BT initiation. Secondly, it was difficult to determine the treatment outcomes from claims data. We were unable to determine the reasons for discontinuation of treatment, which could have included adverse events, lack of efficacy, or even clinical remission. However, compared with the persistent group, no conspicuous decrease in the total medical costs, or increase in the costs of drugs other than BTs in the non-persistent group, were observed, suggesting that a clinical remission may not have been the main reason for the discontinuation of BTs within 12 months. Thirdly, the generalizability of these findings should be approached with caution. As our data were generated from the JMDC database, we cannot rule out the existence of bias towards younger patients. The results may, therefore, not be very representative of the patients with psoriasis aged older than 65 years of age in Japan.

Also, due to our study’s relatively small sample size, we were not able to test determinants of treatment discontinuation. Data from a large UK registry-reported multivariate analysis showed that being female, an active smoker, and – surprisingly – a higher baseline dermatology life quality index were predictors of discontinuation [29]. For second-line biologic therapy it was shown that being female, having multiple comorbidities and a high Psoriasis Area and Severity Index when switching to second-line biologic therapy were general predictors for discontinuation [30]. Finally, we need to acknowledge the differences in baseline covariates across different BTs, in particular with respect to gender. The variation of female patients across different biologic agents might be related to different perceptions of evidence availability in female psoriasis patients who are pregnant or lactating [31]. As women tend to discontinue their treatment more often than men, this is a potential source of a bias.

## Conclusions

Our analysis using claims data of psoriasis patients in Japan revealed an adherence rate for UST that is closer to the results found by the EU studies and higher than the results found by the US studies. On the other hand, the adherence rates for ADL and IFX were found to be closer to the results obtained in the US studies and lower than the results in the EU studies.

Like most other studies have revealed our study also found UST to have the highest adherence rate among all BTs for psoriasis treatment, in both the BT-naïve patient group or the total patient group. The 12-month medical costs after BT initiation for adherent patients may not have increased as much as in the non-adherent patient group. Factors associated with a longer BT adherence period, which may lead to a better clinical outcome and possible cost-savings for patients are expected to be explored in future studies.

## Additional file

Additional file 1: Appendix A. ICD codes for disease to be excluded. Appendix B. Dosage regimen of included BTs. Appendix C. Gap definition (days) of included BTs in base case and sensitivity analysis (SA) 1 and 2. Appendix D. The rates of (number of persistent patients during the 12-month period after BT initiation/number of total patients) calculated in the base case and sensitivity analysis (SA) 1 and 2. (DOCX 23 kb)

## Abbreviations

ADL: Adalimumab
BTs: Biological therapies
CI: Confidence interval
EU: European Union
ICD: International Classification of Diseases
IFX: Infliximab
JMDC: Japan Medical Data Center Co., Ltd.
JPY: Japanese Yen
QoL: Quality of life
RWD: Real-world data
SCK: Secukinumab
US: United States
UST: Ustekinumab
