# Supplementary material for: Persistence rates and medical costs of biological therapies for psoriasis treatment in Japan: a real-world data study using a claims database
Source: BMC Dermatol. 2018 Jul 11;18:5. doi: 10.1186/s12895-018-0074-0 (PMC6042444; doi:10.1186/s12895-018-0074-0)
Supplement: Supplementary file 1 — Appendix A. ICD codes for disease to be excluded. Appendix B. Dosage regimen of included BTs. Appendix C. Gap definition (days) of included BTs in base case and sensitivity analysis (SA) 1 and 2. Appendix D. The rates of (number of persistent patients during the 12-month period after BT initiation/number of total patients) calculated in the base case and sensitivity analysis (SA) 1 and 2. (DOCX 23 kb) [file 12895_2018_74_MOESM1_ESM.docx]

Appendix A. ICD codes for disease to be excluded

| Diseases | ICD codes |
| --- | --- |
| Rheumatoid arthritis | M05 |
|  | M06 |
|  | M315 |
|  | M32 |
|  | M33 |
|  | M34 |
|  | M351 |
|  | M353 |
|  | M360 |
| Inflammatory bowel disease | K50 |
|  | K51 |
|  | K52 |
| Ankylosing spondylitis | M45 |
| Juvenile arthritis | M08 |

Appendix B. Dosage regimen of included BTs

| BTs | Recommended dose | DDD (maintenance) | Available strength/ SKU in Japan market |
| --- | --- | --- | --- |
| Adalimumab (HUMIRA) | Induction: 80 mg at 0 week,  Maintenance: 40 mg every other week | 2.9 mg | 1. Humira SC injection 40 mg Syringe 0.4 mL 2. Humira SC injection 40 mg Syringe 0.8 mL 3. Humira SC injection 80 mg Syringe 0.8 mL 4. Humira SC injection 40 mg Pen 0.4 mL |
| Infiximab (REMICADE) | Induction: 5mg/kg at 0, 2, and 6 weeks,  Maintenance: 5 mg/kg every 8 weeks | 6.25 mg | 1. Remicade intravenous infusion 100 mg |
| Ustekinumab (STELARA) | Induction: 45 mg at 0, 4 weeks,  Maintenance: 45 mg every 12 weeks | 0.54 mg | 1. Stelara SC injection 45 mg Syringe |
| Secukinumab (COSENTYX) | Induction: 300 mg at 0-4 weeks, Maintenance: 300 mg every 4 weeks | 10.71 mg | 1. Cosentyx SC injection 150 mg Syringe 2. Cosentyx SC injection 150mg Pen |

BTs: Biological therapies, SKU: stock-keeping unit, SC: Subcutaneous, mg: Milligrams, kg: Kilograms, ml: Milli-litre

Appendix C. Gap definition (days) of included BTs in base case and sensitivity analysis (SA) 1 and 2

|  | Base case | SA1 | SA2 |
| --- | --- | --- | --- |
| ADL | 60 | 30 | 90 |
| IFX | 60 | 30 | 90 |
| SCK | 60 | 30 | 90 |
| UST | 120 | 90 | 150 |

Adalimumab: ADL, Infliximab: IFX, Secukinumab: SCK, Ustekinumab: UST

# Appendix D. The rates of (number of persistent patients during the 12-month period after BT initiation/number of total patients) calculated in the base case and sensitivity analysis (SA) 1 and 2

|  | Total | | | | BT-naïve | | | |
| --- | --- | --- | --- | --- | --- | --- | --- | --- |
|  | ADL | IFX | SCK | UST | ADL | IFX | SCK | UST |
| Base case | 33.3% | 30.0% | 50.0% | 78.4% | 28.6% | 31.6% | 0.0% | 76.7% |
| SA1 | 16.7% | 0.0% | 50.0% | 67.6% | 21.4% | 0.0% | 0.0% | 66.7% |
| SA2 | 38.9% | 45.0% | 50.0% | 78.4% | 35.7% | 42.1% | 0.0% | 76.7% |

Adalimumab: ADL, Infliximab: IFX, Secukinumab: SCK, Ustekinumab: UST
